# ILCS: An Improved Lightweight Convolution Structure and Mixed Interactive Attention for Steel Surface Defect Classification

**DOI:** 10.1155/2022/7539857

**Published:** 2022-07-18

**Authors:** Yangjun Pei, Mingyang Hou, Qi Han, Tengfei Weng, Yuan Tian, Guorong Chen, Jinyuan Liu, Chen Wu

**Affiliations:** School of Intelligent Technology and Engineering, Chongqing University of Science and Technology, Chongqing 401331, China

## Abstract

The classification method of steel surface defects based on deep learning provides a basis for quality control of industrial steel manufacturing. Due to a large number of interference in the steel production area and the limited computing resources of the edge equipment deployed in the production area, it is a challenge to develop a lightweight model to achieve rapid and accurate classification in the case of limited computing resources. In this article, an improved lightweight convolution structure (LCS) is proposed, which combines the separable structure of convolution and introduces depth convolution and point direction convolution instead of the traditional convolutional module, so as to realize the lightweight of the model. In order to ensure the classification accuracy, spatial attention and channel attention are combined to compensate for the accuracy loss after deep convolution and point direction convolution respectively. Further, in order to improve the classification accuracy, a mixed interactive attention module (MIAM) is proposed to enhance the extracted feature information after LCS. The experimental results show that the recognition accuracy of our method exceeds that of the traditional model, and the number of parameters and the amount of calculation are greatly reduced, which realizes the lightweight of the steel surface defect classification model.

## 1. Introduction

Recently, the defect recognition technology based on traditional machine vision [1, 2] has been applied to the quality inspection of the steel industry, and the automatic detection and classification of surface defects are realized by the machine vision method. However, with the rapid development of the modern steel industry, the steel industry is demanding higher and higher surface quality of steel, and enterprises have more strict requirements for accuracy and recognition effect. Traditional machine vision methods cannot meet the needs of industrial mass production. Considering the above issue, in order to improve the identification accuracy and efficiency of steel surface detection, aiming at guiding production, and further ensuring the quality of steel, scholars have proposed a series of steel surface detection methods [3, 4].

Feature extraction is an important step in steel surface defect recognition. In recent years, feature extraction methods based on different strategies have emerged according to the characteristics of the steel surface. Feature extraction methods mainly include traditional digital vision [5] and deep learning processing methods [6–8].

Traditional digital vision methods usually use shallow features for surface defects, such as color, texture, edge, etc. In complex scenes, traditional methods do not combine multi-feature and multi-scale feature fusion, so the image is not effectively represented. To solve these problems, the deep learning method is applied to steel surface defect detection, which includes steel surface defect recognition, detection [9], segmentation, and other tasks. However, because the speed of a deep neural network is greatly affected by model parameters and calculations, it is not suitable to run on mobile terminals and embedded devices.

Considering the above factors, we design an improved lightweight convolution structure. By constructing a lightweight feature extractor, the number of parameters is reduced and the model is smaller and lighter. In addition, we introduce attention mechanism to further improve the recognition accuracy of complex defects by guiding the network to focus on the defect area. To sum up, we propose an improved lightweight convolution structure and mixed interactive attention method for steel surface defect classification.

Our method has high accuracy and strong robustness. And the most important thing is that the model proposed by us has a small size and is more suitable for edge devices to carry out rapid industrial defect detection. In the feature extraction stage, we use the advantage of depth separable convolution to extract image features quickly and decompose a 3 × 3 convolution kernel into a 3 × 1 convolution kernel and 1 × 3 convolution kernel, which can reduce the number of parameters to be computed. Next, inspired by the CBAM [10] attention module, a mixed interactive attention module MIAM is constructed. The mixed interactive attention module fuses spatial information in the local sensing field, channel information in channel dimension, and rich interactive information in the feature map. In addition, the module enriches the diversity and details of the features and improves the performance of the features.

The main contributions of this article are summarized as follows:

- We propose a novel end-to-end ILCS in order to identify the surface defects of steel. Using attention mechanism, ILCS that combines spatial, channel, and their own interactive information makes the feature information more abundant and effective. And ILCS pays attention to the surface defects of steel itself and weakens the background information.
- We introduce depth convolution and point direction convolution in LCS to replace the traditional convolution. On the basis of greatly reducing the model parameters, our proposed method achieves higher accuracy and faster detection speed.
- We propose a mixed interactive attention model MIAM, which can improve the expressiveness of feature maps while adding a small number of parameters, thereby improving accuracy. And MIAM which is a plug-and-play module can be simply inserted into any other deep learning model.

The rest of this article is organized as follows: Section 2 introduces some related works of this article.  Section 3 introduces our proposed lightweight convolution structure (LCS) for feature extraction, mixed interactive attention model (MIAM) for feature enhancement, and some model details.  Section 4 evaluates our method and compares it with the most advanced method. We conclude our thesis in Section 5.

## 2. Related Works

### 2.1. Convolutional Neural Networks

In recent years, convolutional neural network structure [11] has made great progress in the field of computer vision due to its good performance. LeNet which was the earliest convolutional neural networks was proposed in 1994 [12], and it is a pioneering and innovative achievement completed by Lecun et al. LeNet reduces the amount of calculation compared to ordinary neural networks by developing deep learning feature extraction. Then in 2012, Krizhevsky et al. published AlexNet [11], which further promoted the development of computer vision. AlexNet expanded the idea of LeNet [12] to a larger neural network that can learn more complex object level. Based on the classical structure [11, 12], researchers have proposed many new convolutional neural network structures. Convolutional neural network has become the mainstream method of computer vision.

Szegedy et al. from Google began to seek to reduce the computing cost of a deep learning network and designed GoogleNet [13], which was the first perception architecture. After GoogleNet, ResNet [14] has brought about new changes and is one of the most influential papers in recent years. ResNet has a simple idea, where ResNet supplies the output of two consecutive convolution layers and shunts the input into the next layer. Inspired by the core structure of GoogleNet and ResNet, some new networks are proposed, such as EffNet [15]. They proposed new convolution structures, making the model lighter and significantly reducing the computational burden.

### 2.2. Lightweight Neural Network

With the popularization of deep learning, the volume of neural network is becoming larger and larger, the structure is becoming more and more complex, and the number and depth of network layers are also increasing. Although the prediction effect is improving, the cost of training and prediction is rising, and the demand for hardware resources is also rising. A model with a large amount of parameters and calculation is usually only suitable for servers with strong computing power to train and run, and the model is not suitable for mobile devices or edge devices with limited hardware resources and computing power. So, in the field of deep learning, scholars strive to promote the development of a neural network to miniaturization, while ensuring the accuracy and faster speed. In recent years, researchers put forward lightweight network models such as ShuffleNet [16], NASNet [17], MnasNet [18], MobileNets, and MobileNetV2 [19]. These models make it possible for mobile terminals and embedded devices to run neural network models. MobileNet is more representative in a lightweight neural network. Our network structure borrowed some characteristics of MobileNet series to construct LCS.

## 3. Attention Mechanism for Vision Tasks

Attention mechanism takes the idea of human visual attention. At present, attention mechanism is widely used in nature language processing and image recognition. In 2014, the Google mind team published “recurrent models of visual attention” [20], which used attention mechanism to classify images based on traditional RNN model and achieved good performance. Since then, attention mechanism has been widely used in deep learning tasks. Researchers turned to how to add attention mechanism to convolutional neural network (CNN). ABCNN [21] was an earlier exploratory work of attention in CNN, where three methods were proposed to use attention mechanism in CNN. Reference [21] described how to add attention to CNN where attention was added before convolution in the first method, attention was added in the second method during pooling, and the first and second methods were integrated to realize attention in the third method. These three methods proposed by Yin et al. provide us with a new idea. Then, combining with this idea, many attention modules based on CNN have been proposed in recent years, which contained residual attention networks [22], STN [23], SENet [24], and CBAM [10]. These attention mechanisms have a good performance on CNN. This article combines the idea of channel attention and spatial attention of CBAM [10] to construct our network structure.

## 4. ILCS Module

**Figure 1:**
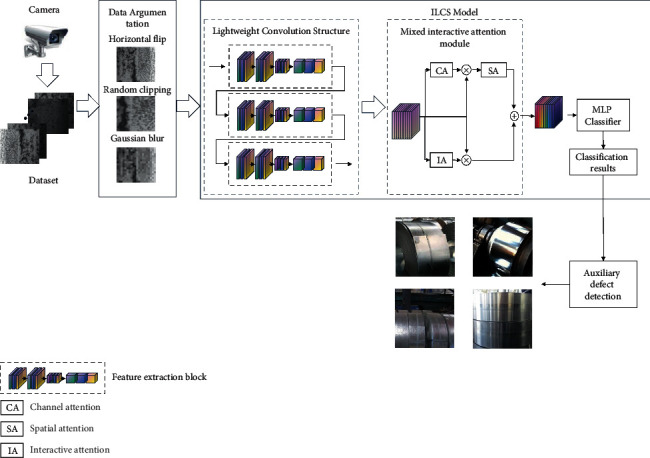
The network architecture of ILCS and flowchart of defect detection of steel, where ⊗ denotes a point-by-point product operation and ⊕ denotes a point-by-point sum operation.

In model training, data augmentation is first performed based on the collected dataset. Then the enhanced dataset is trained by the ILCS model. The network architecture of ILCS consists of a lightweight convolution structure (LCS), a mixed interactive attention model (MIAM), and an MLP classifier. The network architecture is shown in Figure 1. In model testing, the trained model is used to classify defective images and the classification results are used to assist in defect detection.

The proposed architecture takes the steel surface defect image as input, while the output is the defect category label. The size of each defect image is 300 × 300 × 3 (width, height, and channel). The input first generates the feature tensor of size 37 × 37 × 256 by constructing a LCS, where a LCS contains three feature extraction blocks, and the output dimensions are 150 × 150 × 64 in Block 1, 75 × 75 × 128 in Block 2, and 37 × 37 × 256 in Block 3.

In order to further improve the representation ability of feature maps, we will obtain the feature tensor of 37 × 37 × 256 to enhance its features by mixed interactive attention, allowing us to focus on the important features and suppress the unnecessary ones.

Finally, the new feature tensor is converted to a one-dimensional feature vector, and then a fully connected layer is connected with the output. LCS can quickly determine whether an image contains defects and classify the image into the appropriate defect category based on the type of surface defects. The algorithm of ILCS is shown in Algorithm 1.

### 4.1. LCS

**Figure 2:**
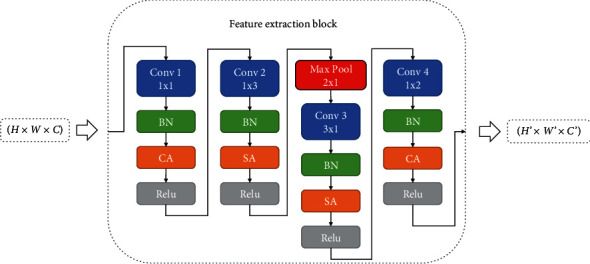
The internal structure of the feature extraction block, where *H*, *W*, *C* represent height, width, and channel, respectively.

The internal structure of the LCS block is shown in Figure 2. Each feature extraction block includes convolution layer (Conv), batch normalization layer (BN), spatial attention (SA), activation layer (ReLU), and channel attention (CA).

#### 4.1.1. Depth Separable Convolutions

The traditional convolutional neural network has been widely used in many fields and has made great achievements in many machine learning projects. But it still has a severe problem which is overspending and mainly reflected in two aspects. The first is the consumption of computing resources and the second is the consumption of time cost. Based on the previous issues, researchers proposed convolution operation, according to two different perspectives of spatial dimension and depth dimension. One is spatial separable convolutions based on spatial perspective, and the other is depthwise separable convolutions based on depth perspective. Inspired by the above two ideas, in this article, we introduce depthwise separable convolution and design our module.

MobileNet [19] converts a standard convolution to a deep separable convolution. The deep separable convolution method of learning spatial characteristics and channel characteristics greatly reduces the number of model parameters. EffNet [15] further divides the depthwise convolution of 3 × 3 in depth separable convolution into convolutions of 1 × 3 and 3 × 1, which greatly reduces the amount of calculation without losing accuracy. Similarly, this article draws on the idea in [25] and designs a 1 × 3 convolution kernel and a 3 × 1 convolution kernel in feature extraction block to replace the large convolution kernel of 3 × 3 and reduce the calculation amount. We use this separation method to make the feature semantic information extracted by convolution focus on the spatial dimension. By using this separation method, edge details of steel surface defects can be learnt.

**Figure 3:**
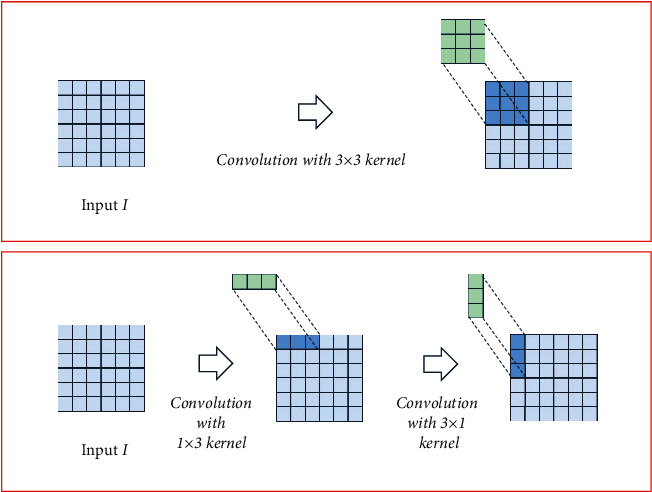
Separation of the depthwise convolution kernel of 3 × 3 into convolution kernels of 1 × 3 and 3 × 1.

In order to calculate the number of parameters, the characteristic tensor of the input block in LCS is *H* × *W* × *C*_in_ and the tensor of output feature is *H* × *W* × *C*_out_, where *H*, *W*, *C*_in_, and *C*_out_ represent height, width, channel of input, and channel of output, respectively. According to the above description, we convert ordinary convolution to deep separable convolution [19], which includes depthwise convolution (DWC) kernel *K* × *K* × 1 and pointwise convolution (PWC) kernel 1 × 1 × *C*_in_. As shown in Figure 3, we decompose DWC into convolution cascades of 1 × *K* × 1 kernel and *K* × 1 × 1 kernel to reduce the number of parameters. For example, we can separate the convolution kernel of 3 × 3 into convolution kernels of 1 × 3 and 3 × 1. Two PWC of kernels 1 × 1 × *C*_in_ and 1 × 2 × *C*_in_ are used in our structure, and their positions are before and after the two cascaded DWC. The original convolution is compared with our number of structural parameters, which is expressed as follows:

(1) $$\begin{array}{c} \frac{\left(C_{in}\times C_{out}\right)+\left(K\times C_{in}\right)+\left(K\times C_{in}\right)+\left(2\times C_{in}\times C_{out}\right)}{K\times K\times C_{in}\times C_{out}}=\frac{2}{K\times C_{\text{out }}}+\frac{3}{K\times K}, \end{array}$$

where *K* × *K* × *C*_in_ × *C*_out_ is the number of parameters of original convolution, *K* × *K* × 1 × *C*_in_ is the number of parameters of DWC, and the number of parameters of ILCS equals *K* × 1 × 1 × *C*_in_ plus 1 × *K* × 1 × *C*_in_. Then we add two PWCs where the number of parameters of two PWCs is 1 × 1 × *C*_in_ × *C*_out_ and 1 × 2 × *C*_in_ × *C*_out_. From equation (1), we know that the LCS can greatly reduce the number of model parameters and improve the calculation speed.

#### 4.1.2. Lightweight Convolution Structure

Inspired by MobileNet [19] and EffNet [15], we introduce and combine depth convolution and point direction convolution in order to replace the traditional convolution to construct a basic feature extractor called LCS.

This LCS architecture consists of four convolution layers, four batch normalization layers, four ReLU layers, two spatial attention (SA), two channel attention (CA), and one pooling layer. The LCS is shown in Figure 2. The detailed configuration of individual layers/modules in the LCS is shown in Table 1 for defect classification on the NEU benchmark dataset.

In Block *i*, Conv1 and Conv4 are for channel feature extraction and Conv2 and Conv3 are for spatial feature extraction. And after each convolution operation, we connect a BN layer to prevent the gradient from disappearing and speed up the network convergence in this block. First of all, we use 1 × 1 convolution operation to achieve reduced dimensions for the number of channels and rectified linear activation [13], not only for the convenience of cascade network, but also for adapting multi-channel image input. Then we use a convolution kernel of 1 × 3 and a convolution kernel of 3 × 1 to replace the ordinary convolution kernel of 3 × 3, and finally we use the convolution kernel of 1 × 2 to get the final characteristic graph.

The essence of neural network is to learn the distribution of data, but when we build the network model, we find that the generalization ability of the model is poor.

In order to solve this problem, we do batch normalization (BN) after each convolution operation, where BN introduces normalized activation into the LCS block. This method ensures that when the LCS is trained, BN' layers can continue learning on input distributions that exhibit less internal covariate shift, thus accelerating the training [26]. And BN also can enhance the generalization ability of the model. The batch normalizing transform formula is as follows:

(2) $$\begin{array}{c} y=\frac{x-E\left[x\right]}{\sqrt{Var\left[x\right]+\int }}*\gamma +\beta , \end{array}$$

where E[*x*] is mini-batch mean, Var[*x*] is mini-batch variance, and *γ* and *β* are scale and shift, respectively, and they are learnable parameter vectors.

**Figure 4:**
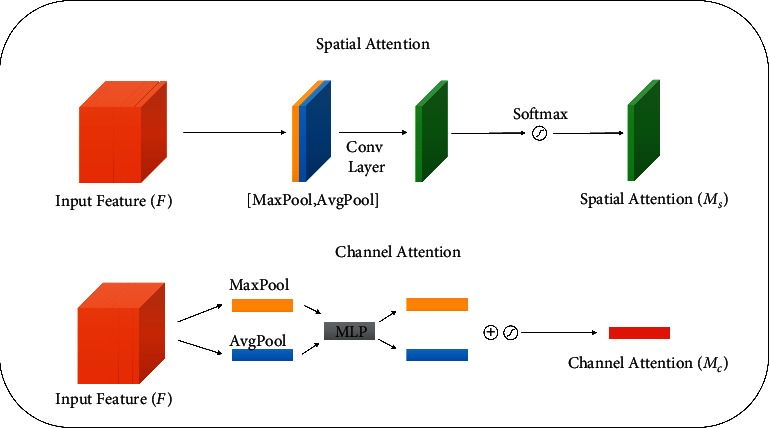
Spatial and channel attention module.

In order to improve the expressiveness and accuracy of spatial dimension and channel dimension, we add spatial attention (SA) shown in Figure 4 to focus on region-of-interest after Conv2 and Conv3, which can effectively enhance regional characteristics. Channel attention (CA) is added to focus on channel after Conv1 and Conv4, which can effectively enhance the weight of channel characteristics and the spatial perception ability. Otherwise, convolution is usually followed by a ReLU nonlinear activation function. Based on the idea of MobileNet [19], we use ReLU6 in MobileNet. ReLU6 is an ordinary ReLU, but the maximum output is limited to 6, which is to prevent large precision loss caused by excessive activation output value. In our experiment, we found that ReLU6 has a good performance in our network. The formula for ReLU6 is as follows:

(3) $$\begin{array}{c} \text{ReLU}6\left(x\right)=\min\left(\max\left(0,x\right),6\right). \end{array}$$

### 4.2. MIAM

#### 4.2.1. Spatial Attention Module

In order to highlight the effective features of defect images in spatial information, we add SA in LCS and MIA. Specifically, through average pooling of spatial dimension and maximum pooling of spatial dimension operation, two feature maps are obtained. Two feature maps are concatenated by channel dimension, subsequently. And the merged feature map is inputted into the convolution layer for convolution. Then spatial attention map  **M**_**s**_(**F**) is further formed, which is as follows:

(4) $$\begin{array}{c} M_{s}\left(F\right)=\sigma \left(f^{3\times 3}\left(\left[\text{AvgPool}\left(F\right);\text{MaxPool}\left(F\right)\right]\right)\right), \end{array}$$

(5) $$\begin{array}{c} \sigma \left(x\right)=\frac{1}{1+e^{-x}}, \end{array}$$

where **F** ∈ *ℝ*^*H*×*W*×*C*^ is the feature map obtained from the LCS feature extractor, *H* and *W* are the height and width of the feature map, respectively, and *C* is the number of channels. **F** can be expressed as **F**=[**f**_1_, **f**_2_,…, **f**_*C*_], where  **f**_*i*_ ∈ *ℝ*^*H*×*W*^ is each feature map, *f*^3×3^ represents a convolution operation with the filter size of 3 × 3, and *σ* denotes the sigmoid function.

#### 4.2.2. Channel Attention Module

In order to highlight the effective features of defect images in channel information, we add CA in LCS and MIA. The structure of CA is shown in Figure 4. Different from spatial attention, CA obtaining two feature vectors are computed by averaging pooling and maximum pooling of channel respectively. Then two feature vectors are linearly transformed by MLP, finally, and two feature vectors are fused to obtain the channel attention **M**_**c**_(**F**). The formulas of **M**_**c**_(**F**) and *MLP* are as follows:

(6) $$\begin{array}{c} M_{c}\left(F\right)=\sigma \left(\text{MLP}\left(\text{AvgPool}\left(F\right)\right)+\text{MLP}\left(\text{MaxPool}\left(F\right)\right)\right), \end{array}$$

(7) $$\begin{array}{c} \text{MLP}\left(x\right)=W_{1}\left(W_{0}\left(x\right)\right), \end{array}$$

where a multiple layer perception (MLP) is implemented by two fully connected layers, and AvgPool and MaxPool represent global average pooling and global maximum pooling, respectively, to obtain global information for each channel.

#### 4.2.3. Interactive Attention

According to the biological visual interaction mechanism [27], we construct an interactive attention block, which enriches the feature details of the attention area. In interactive attention, the input feature map is transformed into **F**^**T**^ through transpose operation, and then **F**^**T**^ is used to multiply the original input feature map **F** point-by-point to obtain new self-interactive feature information, so as to enrich the original feature map.

**Figure 5:**
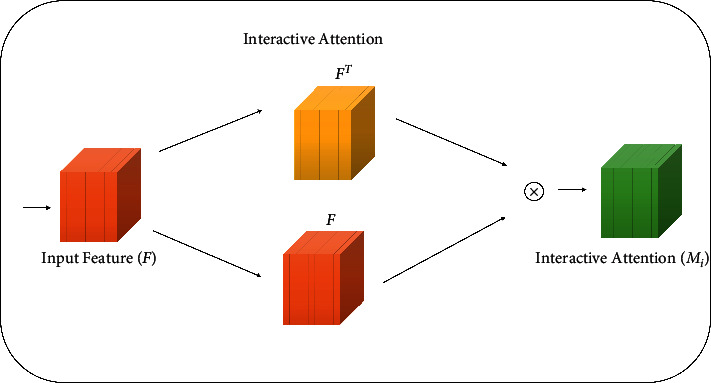
Interactive attention module.

The architecture of the interactive attention block is shown in Figure 5. The formula is as follows:

(8) $$\begin{array}{c} M_{i}\left(F\right)=F\cdot F^{T}, \end{array}$$

where  ·  is point-by-point product operation and **T** refers to matrix transpose operation.

#### 4.2.4. Mixed Interactive Attention Module

**Figure 6:**
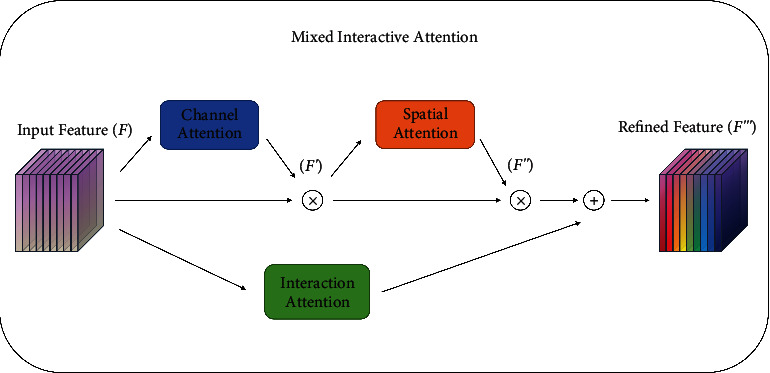
Mixed interactive attention module.

A MIAM can fuse spatial information **M**_**s**_(**F**) with channel information **M**_**c**_(**F**) and rich interactive information **M**_**i**_(**F**). The mixed interactive attention module is shown in Figure 6.

First, the channel information **M**_**c**_(**F**) can be obtained by equation (6). Then channel information **M**_**c**_(**F**) times original feature map **F** to obtain a new feature map *F*′, which can enhance channel information, and the formula is as follows:

(9) $$\begin{array}{c} F^{\prime }=M_{c}\left(F\right)*F. \end{array}$$

Secondly, from *F*′, the spatial information **M**_**s**_(*F*′) can be obtained by (4). Then spatial information **M**_**s**_(*F*′) times original feature map *F*′ to obtain a new feature map *F*^″^, which can enhance spatial information, and the formula is as follows:

(10) $$\begin{array}{c} F^{\prime \prime }=M_{s}\left(F^{\prime }\right)*F^{\prime }. \end{array}$$

Finally, after obtaining *F*^″^ from equation (10), interactive information **M**_**i**_(**F**) adds feature map *F*^″^ to obtain a new feature map *F*^‴^, which combines spatial, channel, and their own interactive information. The feature information is enriched and effective, and the formula is as follows:

(11) $$\begin{array}{c} F^{\prime \prime \prime }=F^{\prime \prime }+M_{i}\left(F\right). \end{array}$$

### 4.3. Integrated Models and Classifier

According to our integration model, a general framework of the ILCS module is shown in Figure 1. We use LCS to extract a series of feature maps for the input image. Then, according to MIAM, we use the cascade module of spatial attention, channel attention, and interaction attention to enhance the representation ability of feature maps. Finally, a multiple layer perception classifier (MLP  classifier) is implemented by two fully connected layers to classify defects and obtain the classification results.

## 5. Experiments

### 5.1. Dataset

We conduct experiments on the NEU dataset, consisting of 6 classes defects, such as rolled-in scale (RS), patches (Pa), crazing (Cr), pitted surface (PS), inclusion (In), and scratches (Sc) and contains 1800 299 × 299 grayscale images. Each class has 300 samples.

### 5.2. Implementation Details

We implement our method by use of PyTorch framework. For comparison, we add a MIAM module of ILCS to original baselines which include ResNet [14], EffNet [15], and MobileNet [19].

Similar to prior work, in the NEU dataset, we use 70% images as training dataset and 30% images as test dataset. In order to improve the accuracy of the result and speed up the convergence of ILCS, all images are normalized before they are introduced in to ILCS. The mean of all pixels in all images in the NEU dataset is 0.4 and the variance is 0.2.

We train ILCS using the optimal hyper-parameter configuration network, and set a mini-batch of 16 on GTX 1060 GPU. The loss function is cross-entropy loss.

### 5.3. Experimental Results

In this article, firstly, original baselines including ResNet, EffNet, and MobileNet are trained and tested in the NEU dataset. Secondly, original baselines with MIAM are trained and tested again to evaluate the effectiveness of MIAM. Finally, ILCS runs on the NEU dataset and experimental results are compared in Table 2, and the Top-1 acc. and Top-5 acc. are obtained by Algorithm 2.

**Figure 7:**
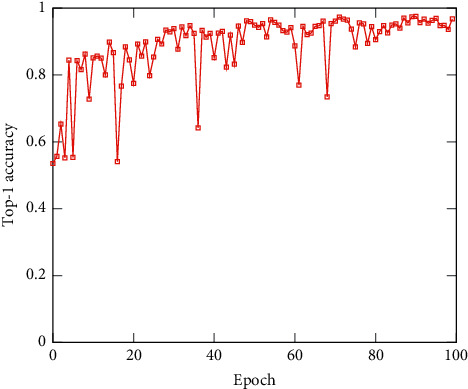
The classification accuracy of the test set of the network.

**Figure 8:**
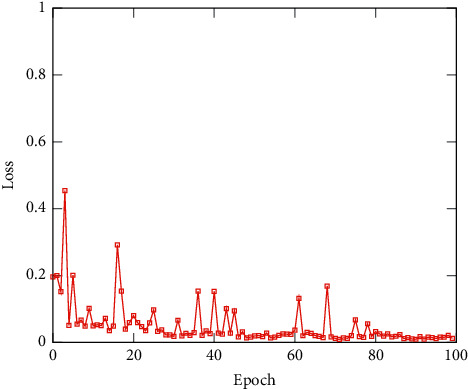
The classification loss of the test set of the network.

To validate the performance of ILCS, we experimentally analyze the effects of different models combined with our methods using Paramms (M) and FLOPs (G) [19] to measure results, and the classification accuracy and loss of the test set of our network are shown in Figures 7 and 8.

From Tables 2 and 3, we can clearly see that baselines combined with our methods can improve model accuracy without limiting model performance.

The EffNet + MIAM achieves a 1.02% % improvement in terms of Top-1 accuracy over the EffNet, but only 0.01 M more parameters and 0.01 G more FLOPs. Also, the MobileNet + MIAM has an improvement of 0.47% % over the MobileNet, but only 0.01 M more parameters and 0.01 G more FLOPs. Finally, compared with the above model, parameters of ILCS are 2.24 M and FLOPs are 4.26 G. Under the same parameter number or lower parameter number structure, we can achieve higher accuracy. In the case of a small increase in parameters, our method outperforms the primitive baseline.

### 5.4. Model Visualization

To understand the ability of the ILCS about paying attention to the defect area, we use the heat map to visualize the attention map of each type of defect image, which is a common method of attention visualization.

**Figure 9:**
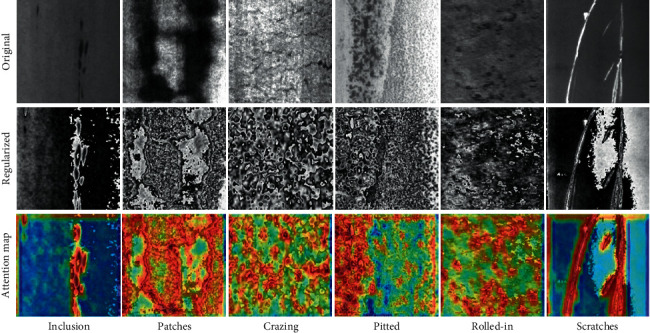
Image attention visualization of six surface defects in NEU surface defect database.

In the visualization examples shown in Figure 9, stronger attention areas are covered by the redder the color, inversely the bluer the color. It can be seen from the figure that our attention method has an obvious effect on linear and block defects, such as “Inclusion,” “Patches,” and “Scratches.” This ability to pinpoint attention areas makes our approach more valuable for classification, detection, segmentation, etc.

## 6. Conclusion and Future Work

In this article, a simple and effective block is proposed to further explore the effectiveness of attention mechanism in the classification of steel surface defects, that is the interactive attention block for the classification of steel surface defects, which effectively enhances the attention weight of defect areas.

Based on the fast feature extraction of LCS, we suggest that the network should further pay attention to channel information, spatial information, and its own interactive information, so we add the interactive attention block to space and channel attention to form MIAM. The results show that our method can improve the attention of the backbone network to the defect area, so as to improve the identification accuracy of the backbone network.

The image classification results in the NEU dataset show that the interactive attention block in this article improves the defect classification accuracy of different CNN models based on lightweight backbone networks, and only a small amount of calculation parameters is added. The visualization results show that the interactive attention block can help the model to focus on most types of defects.

In addition, this block can be combined with the backbone network of the lightweight model to achieve rapid processing, so it can be used for industrial production quality inspection and further realize the automation of steel production.

Finally, our model has defects in many aspects. For example, the current model only classifies the defect image but does not locate the defect region. In addition, for industrial applications, complex industrial background noise images should be considered to suppress the background noise, which our model has not done. In the future, our research direction will focus on defect location and industrial complex background noise suppression and modify and improve our model to achieve higher accuracy and efficiency in industrial defect detection tasks.

## Figures and Tables

**Algorithm 1:**
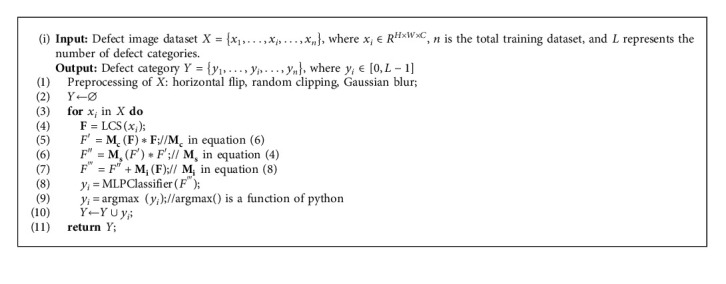
The classification model based on ILCS.

**Algorithm 2:**
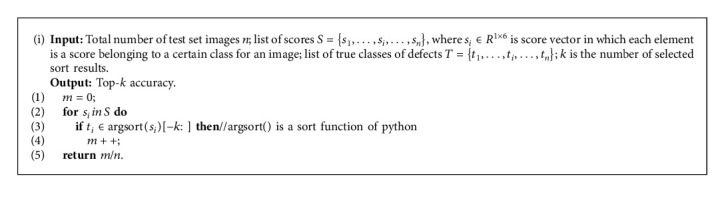
Top-*k* accuracy.

**Table 1:** The configuration of each layers in the LCS.

Layers	Output size	Filter size
Input	*H* × *W* × *C*××	—

Block 1	150 × 150 × 64	32 × 1 × 1
		32 × 1 × 3
		32 × 3 × 1
		64 × 1 × 2

Block 2	75 × 75 × 128	64 × 1 × 1
		64 × 1 × 3
		64 × 3 × 1
		128 × 1 × 2

Block 3	37 × 37 × 256	128 × 1 × 1
		128 × 1 × 3
		128 × 3 × 1
		256 × 1 × 2

MIA	37 × 37 × 256	—

MLP	1 × 1 × 6	—

**Table 2:** Top-1 and top-5 test accuracy (%) of deeper networks on the NEU dataset.

	Top-1 acc. (%)	Top-5 acc. (%)
ResNet	95.09	100.00
ResNet + MIAM	**96.36**	**100.00**
EffNet	94.81	100.00
EffNet + MIAM	**95.83**	**100.00**
MobileNet	95.57	100.00
MobileNet + MIAM	**96.04**	**100.00**
ILCS (ours)	**97.50**	**100.00**

The bold values are the ablation experimental results of our attention block and the Top1 accuracy and top5 accuracy of our ILC in the dataset.

**Table 3:** Params (M) and FLOPs (G) of module in the NEU dataset.

	Params (M)	FLOPs (G)
ResNet	25.56	65.78
ResNet + MIAM	**25.57**	**65.79**
MobileNet	2.23	4.70
MobileNet + MIAM	**2.24**	**4.71**
EffNet	2.21	8.35
EffNet + MIAM	**2.22**	**8.36**
ILCS (ours)	**2.24**	**4.26**

Bold values are the ablation experimental results of our attention block and the Params (M) and FLOPs (G) of our ILC in the dataset. Our method only increases a small number of parameters, but greatly improves the accuracy.

## Data Availability

Previously reported data were used to support this study and are available at https://doi.org/10.1016/j.apsusc.2013.09.002. These prior studies (and datasets) are cited at relevant places within the text as references [1].
